# Cervical Cancer Screening Rates Among Rural and Urban Females, From 2019 to 2022

**DOI:** 10.1001/jamanetworkopen.2024.17094

**Published:** 2024-06-14

**Authors:** Tyrone F. Borders, Amanda Thaxton Wiggins

**Affiliations:** 1 Center for Health Services Research, University of Kentucky, Lexington; 2 College of Nursing, University of Kentucky, Lexington

## Abstract

**Importance:**

Little nationally representative research has examined Papanicolaou testing rates from before the pandemic in 2019 through the COVID-19 pandemic in 2022. Papanicolaou testing rates among rural females are a concern as they have historically had lower screening rates than their urban counterparts.

**Objective:**

To examine the receipt of a Papanicolaou test in the past year among US females overall and females residing in rural and urban areas in 2019, 2020, and 2022.

**Design, Setting, and Participants:**

This repeated cross-sectional study used data from 3 years of the Health Information National Trends Survey (HINTS), a nationally representative survey that asks respondents about cancer screenings, sources of health information, and health and health care technologies. Study participants were females aged 21 to 65 years. Individuals who received a Papanicolaou test more than 1 to 3 years prior to a HINTS interview were excluded as they were likely not due for a Papanicolaou test.

**Exposures:**

Survey year (2019, 2020, and 2022) and rural or urban residence were the main exposure variables.

**Main Outcomes and Measures:**

Self-reported receipt of a Papanicolaou test within the past year.

**Results:**

Among the 188 243 531 (weighted; 3706 unweighted) females included in the analysis, 12.5% lived in rural areas and 87.5% in urban areas. Participants had a mean (SE) age of 43.7 (0.27) years and were of Hispanic (18.8%), non-Hispanic Asian (5.2%), non-Hispanic Black (12.2%), non-Hispanic White (59.6%), or non-Hispanic other (4.1%) race and ethnicity. In 2022, unadjusted past-year Papanicolaou testing rates were significantly lower among rural vs urban residents (48.6% [95% CI, 39.2%-58.1%] vs 64.0% [95% CI, 60.0%-68.0%]; *P* < .001). Adjusted odds of past-year Papanicolaou testing were lower in 2022 than 2019 (odds ratio, 0.70; 95% CI, 0.52-0.95; *P* = .02).

**Conclusions and Relevance:**

This repeated cross-sectional study found that past-year Papanicolaou testing rates were lower in 2022 than 2019, pointing to a need to increase access to screenings to prevent an uptick in cervical cancer incidence. Rural-vs-urban differences in 2022 indicate a need to specifically target rural females.

## Key Points

**Question:**

Did Papanicolaou testing rates change among rural and urban females during the COVID-19 pandemic?

**Findings:**

In this repeated cross-sectional survey of 188 243 531 US females, the adjusted odds of receiving a Papanicolaou test in the past year were lower in 2022 than 2019. In 2022, 48.6% of rural and 64.0% of urban residents received a Papanicolaou test in the past year.

**Meaning:**

Findings from this study suggest a need to rapidly increase access to cervical cancer screenings to prevent an uptick in cervical cancer incidence in the future, especially among rural females.

## Teaser

This cross-sectional study assesses the delays and disruptions in Papanicolaou testing associated with the COVID-19 pandemic among female adults living in rural and urban areas.

## Introduction

Emerging evidence indicates that the COVID-19 pandemic disrupted the receipt of recommended screenings for the detection of common cancers, including cervical cancer. Research using Behavioral Risk Factor Surveillance System data found that rates of past-year cervical cancer screenings decreased from 58% in 2018 (prepandemic) to 52% in 2020.^1^ Data from an electronic health records system showed that after the national COVID-19 public health emergency was declared, cervical cancer screenings decreased 94%, with an estimated 40 000 screenings missed or delayed between March and June 2020.^2,3^ More recently, a 2021 study of the National Breast and Cervical Cancer Early Detection Program, which delivers cancer screenings for individuals with low income or inadequate insurance, found that cervical cancer screenings were much lower in spring 2020 than the previous 5-year mean.^4^ Although prior research provides some evidence that cervical cancer screenings decreased in the early stages of the pandemic, no nationally representative research to date has compared the rates between the prepandemic period and the pandemic through 2022.

Delays in cervical cancer screening are of particular concern for rural residents given that they had higher cervical cancer incidence and mortality rates than urban residents prior to the pandemic.^5,6,7,8^ Moreover, prior studies have shown that rural residents have lower rates of cervical cancer screening than urban residents.^9,10^ Some studies suggest that the pandemic contributed to a reduction in access to cervical and other cancer screenings.^11,12^ A survey of personnel working in Medicare-designated rural health clinics across the US reported that the availability of both Papanicolaou and human papillomavirus (HPV) testing decreased from before the pandemic to March 2020.^11^ A qualitative study of primary care clinic administrators and clinicians conducted between August 2020 and April 2021 identified several common (eg, personnel shortages and less emphasis on primary care services) and different (eg, more restrictive COVID-19 testing policies at rural clinics) barriers to cancer screening among rural and urban clinics.^12^

Papanicolaou tests are an important component of the US Preventive Services Task Force (USPSTF) guidelines for cervical cancer screenings. The USPSTF recommends Papanicolaou testing every 3 years for individuals aged 21 to 29 years and either Papanicolaou testing every 3 years, high-risk HPV testing every 5 years, or a combination of the 2 tests every 5 years among individuals aged 30 to 65 years.^13^ Although there is an option under these recommendations to have a high-risk HPV test instead, a study based on insurance claims data for females 30 to 65 years of age found the Papanicolaou test to be the most common form of cervical cancer screening.^14^

In this study, we used nationally representative data from the National Cancer Institute (NCI) Health Information National Trends Survey (HINTS) to examine the receipt of a Papanicolaou test in the past year among females overall and females residing in rural and urban areas in 2019, 2020, and 2022. We examined past-year Papanicolaou tests over these 3 years because 2019 marked the year prior to the COVID-19 pandemic in the US, 2020 marked an increase in cases and deaths, and 2022 began with a sharp increase followed by a gradual reduction in the number of cases and deaths.^15^

## Methods

### Study Design

A repeated cross-sectional study was conducted using data from the HINTS 5 cycle 3 (2019), HINTS 5 cycle 4 (2020), and HINTS 6 (2022). HINTS is a nationally representative survey conducted by the NCI about adults’ awareness and receipt of cancer screenings, sources of health information, and use of health and health care technologies.^16^ More information about HINTS is provided on the NCI website.^17^ In accordance with the Common Rule, this study was exempt from ethics review and the informed consent requirement because the HINTS data are deidentified and available publicly for download. We followed the Strengthening the Reporting of Observational Studies in Epidemiology (STROBE) reporting guideline.^18^

Eligibility criteria for the present analysis were self-reported female sex at birth and age of 21 to 65 years. Exclusion criterion was receipt of a Papanicolaou test more than 1 to 3 years prior to the HINTS interview because these individuals would not have been due for a Papanicolaou test according to USPSTF guidelines (unless they had received a high-risk HPV test or combined Papanicolaou test and high-risk HPV test within the past 5 years). Of note, the 2021 HINTS did not include a Papanicolaou test question; thus, no 2021 data were available for analysis.

### Outcomes

The outcome of interest was whether a participant received a Papanicolaou test within the past year. We focused on a Papanicolaou test within the past year because this time frame could be more sensitive to disruptions related to the pandemic. The HINTS questionnaire included a single item about the receipt of a Papanicolaou test, which was worded as follows: “How long ago did you have your most recent Pap test to check for cervical cancer?” Response options were as follows: (1) a year ago or less; (2) more than 1, up to 2 years ago; (3) more than 2, up to 3 years ago; (4) more than 3, up to 5 years ago; (5) more than 5 years ago; and (6) I have never had a Papanicolaou test. Because females who received a Papanicolaou test in accordance with USPSTF guidelines (options 2-3) were excluded from this study, the dependent variable was dichotomized as whether a female between the ages of 21 and 65 years received a Papanicolaou test in the past year (option 1) vs did not receive a Papanicolaou test in the past year (options 4-6).

### Exposures and Covariates

The main exposure variables were rural or urban residence, as defined by the US Office of Management and Budget definitions of nonmetropolitan and metropolitan counties,^19^ and the survey calendar years 2019, 2020, and 2022. Other sociodemographic characteristics were age, self-reported race and ethnicity (Hispanic; non-Hispanic Asian [hereafter Asian]; non-Hispanic Black [hereafter Black]; non-Hispanic White [hereafter White]; and non-Hispanic other [hereafter other], including non-Hispanic American Indian or Alaskan Native, non-Hispanic Native Hawaiian or Other Pacific Islander, and non-Hispanic multiple races), spouse or partner status (partnered vs nonpartnered), health insurance status (insured vs uninsured), and educational level (less than high school, high school graduate, some college, and college graduate or higher). Race and ethnicity were included in this study to test for racial and ethnic differences.

### Statistical Analysis

Weighted frequency distributions were used to summarize the sociodemographic characteristics. The Rao-Scott χ^2^ test was used to examine rural vs urban differences in Papanicolaou testing within each survey year. Weighted logistic regression was used to test for changes over time in the odds of past-year Papanicolaou testing. Both unadjusted and adjusted models were examined, each including an interaction term between study year and rural or urban geographic location; the adjusted model also included sociodemographic characteristics. We evaluated the relative excess risk due to interaction to examine the potential additive effect of the interaction.^20^ Missing data were handled using listwise deletion.

All data analyses were conducted in SAS, version 9.4 (SAS Institute Inc) using weighted survey methods (PROC SURVEYFREQ, PROC SURVEYLOGISTIC) with replicate weights. A 2-sided *P* = .05 indicated statistical significance.

## Results

Among the 188 243 531 (weighted; 3706 unweighted) females included in the analysis, 12.5% (432 unweighted) resided in a rural area and 87.5% (3374 unweighted) resided in an urban area. Participants had a mean (SE) age of 43.7 (0.27) years. Regarding race and ethnicity, 5.2% of participants self-identified as Asian, 12.2% as Black, 18.8% as Hispanic, 59.6% as White, and 4.1% as other race and ethnicity. A majority of participants (55.8%) had a spouse or partner and had health insurance (90.4%). Regarding educational level, 5.8% of participants had less than a high school diploma, whereas 39.1% had some college education. Participant characteristics were similar across each of the survey years (Table 1).

**Table 1.** Descriptive Summary of Participant Characteristics Across Study Years^a^

Characteristic	2019		2020		2022	
	Unweighted No.	Weighted No. (%) [95% CI]	Unweighted No.	Weighted No. (%) [95% CI]	Unweighted No.	Weighted No. (%) [95% CI]
Total participants	1256	62 402 371	948	65 521 323	1502	60 319 838
Residence						
Rural	131	7 820 971 (12.5) [9.1-16.0]	103	8 656 330 (13.2) [8.8-17.6]	198	7 145 267 (11.8) [9.5-14.2]
Urban	1125	54 581 399 (87.5) [84.0-90.9]	845	56 864 993 (86.8) [82.4-91.2]	1304	53 174 571 (88.2) [85.8-90.5]
Age, y						
21-29	148	12 270 811 (19.7) [16.0-23.3]	114	13 226 115 (20.2) [16.7-23.7]	172	10 872 063 (18.0) [14.6-21.4]
30-39	243	10 778 454 (17.3) [14.6-20.0]	159	11 922 034 (18.2) [14.3-22.1]	302	12 185 852 (20.2) [7.0-23.4]
40-49	237	14 313 159 (22.9) [19.3-26.6]	213	16 478 311 (25.1) [20.7-29.6]	285	12 945 125 (21.5) [17.8-25.2]
50-59	372	17 977 142 (28.8) [24.6-33.0]	260	15 589 857 (23.8) [19.8-27.8]	412	15 847 316 (26.3) [22.7-29.8]
60-65	256	7 062 805 (11.3) [10.1-12.6]	202	8 305 005 (12.7) [10.9-14.4]	331	8 469 482 (14.0) [12.4-15.7]
Race and ethnicity^b^						
Asian	54	3 062 703 (5.1) [3.1-7.2]	36	3 612 066 (5.7) [2.8-8.6]	66	2 853 112 (4.8) [3.1-6.5]
Black	219	7 207 188 (12.1) [9.7-14.4]	143	7 583 779 (12.0) [9.0-14.9]	287	7 528 592 (12.7) [10.6-14.8]
Hispanic	190	10 779 726 (18.0) [14.9-21.2]	178	7 583 779 (19.3) [16.1-22.5]	307	11 366 217 (19.1) [16.3-22.0]
White	664	36 687 536 (61.6) [58.7-64.6]	513	37 822 513 (59.8) [56.3-63.2]	744	34 033 128 (57.3) [54.7-59.9]
Other^c^	64	1 902 848 (3.2) [1.6-4.8]	35	2 043 607 (3.2) [1.7-4.7]	53	3 595 599 (6.1) [3.6-8.5]
Spouse or partner status						
Partnered	673	34 820 881 (56.1) [53.3-58.9]	513	35 614 705 (54.9) [50.6-59.2]	774	33 848 125 (56.5) [53.7-59.3]
Nonpartnered	579	27 296 092 (43.9) [41.1-46.7]	422	29 283 028 (45.1) [40.8-49.4]	720	26 077 841 (43.5) [40.7-46.3]
Health insurance status						
Insured	1157	57 053 584 (92.2) [89.7-94.7]	867	58 240 415 (89.2) [86.2-92.2]	1335	53 964 643 (89.9) [86.8-92.9]
Uninsured	83	4 799 961 (7.8) [5.3-10.3]	73	7 062 077 (10.8) [7.8-13.8]	159	6 094 449 (10.1) [7.1-13.2]
Educational level						
<High school	68	3 781 019 (6.1) [4.1-8.0]	49	3 775 597 (5.8) [3.3-8.3]	95	3 247 799 (5.4) [3.7-7.1]
High school graduate	191	12 696 849 (20.3) [17.7-23.0]	157	12 688 149 (19.6) [15.6-23.5]	263	11 259 973 (18.7) [16.4-21.0]
Some college	357	24 795 580 (39.7) [36.8-42.7]	250	24 888 525 (38.4) [34.8-41.9]	406	23 644 647 (39.3) [36.7-41.8]
≥College graduate	639	21 121 670 (33.9%) [31.6-36.1]	476	23 501 283 (36.7%) [34.6-38.7]	734	22 084 363 (36.6%) [34.6-38.7]

^a^ Based on nonmissing data for Papanicolaou test question.

^b^ Race and ethnicity were self-reported and obtained from the Health Information National Trends Survey data.

^c^ Other includes non-Hispanic American Indian or Alaskan Native, non-Hispanic Native Hawaiian or Other Pacific Islander, and non-Hispanic multiple races.

In the bivariate analysis (Table 2 and Figure), the weighted past-year Papanicolaou testing rates in 2022 were significantly lower among rural than urban females (48.6% [95% CI, 39.2%-58.1%] vs 64.0% [95% CI, 60.0%-68.0%]; *P* < .001). No significant differences were observed between rural and urban residents in previous years.

**Table 2.** Past-Year Papanicolaou Test by Geographic Residence Across Study Years

Past-year Papanicolaou test	Rural		Urban		OR (95% CI)	Rao-Scott χ^2^	*P* value
	No./Weighted No.	% (95% CI)	No./Weighted No.	% (95% CI)			
2019							
Yes	85/5 326 194	68.1 (57.9-78.3)	828/39 110 099	71.7 (67.2-76.1)	0.84 (0.52-1.38)	0.48	.49
No	46/2 494 777	31.9 (21.7-42.1)	297/15 471 300	28.3 (23.9-32.8)			
2020							
Yes	66/4 943 988	57.1 (42.7-71.5)	601/39 896 714	70.2 (65.5-74.9)	0.57 (0.29-1.10)	3.59	.058
No	37/3 712 342	42.9 (28.5-57.3)	244/16 968 279)	29.8 (25.1-34.5)			
2022							
Yes	99/3 474 510	48.6 (39.2-58.1)	844/34 010 101	64.0 (60.0-68.0)	0.53 (0.36-0.79)	11.38	<.001
No	99/3 670 757	51.4 (41.9-60.8)	460/19 164 470	36.0 (32.0-40.0)			

Abbreviation: OR, odds ratio.

**Figure.**
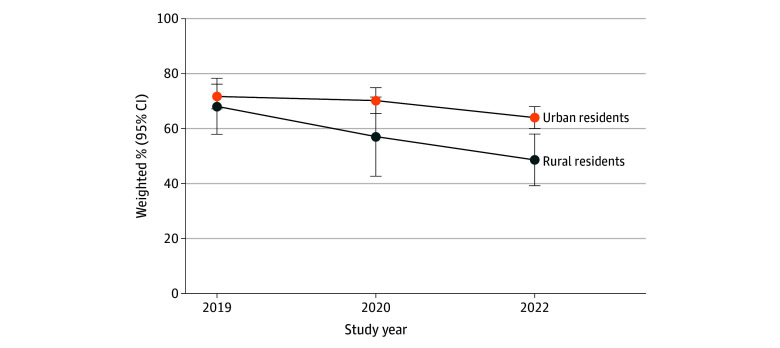
Past-Year Papanicolaou Test by Geographic Residence Across Study Years Error bars represent 95% CIs.

In the weighted logistic regression analysis (Table 3), there were no differences in the rates of change over time in odds of Papanicolaou testing between rural and urban females (*F* = 1.1 [*P* = .33] for unadjusted and *F* = 1.2 [*P* = .30] for adjusted interaction terms). Based on 2019 and 2022 survey years, the relative excess risk due to interaction was −0.06 (95% CI, −0.51 to 0.40) in the unadjusted model and 0.10 (95% CI, −0.41 to 0.62) in the adjusted model, suggesting little to no interaction between survey year and rural or urban residence, consistent with the results found when using the interaction term. While rural residents demonstrated a significantly lower past-year Papanicolaou screening rate in the bivariate analysis, the main outcome for rural residence was not significant in the unadjusted or adjusted models. Comparing survey years, the odds of Papanicolaou testing did not differ between 2019 and 2020, but the odds were approximately 30% lower in 2022 than 2019 in both the unadjusted model (odds ratio [OR], 0.70; 95% CI, 0.53-0.93; *P* = .01) and adjusted model (OR, 0.70; 95% CI, 0.52-0.95; *P* = .02).

**Table 3.** Weighted Logistic Regression Modeling Receipt of Past-Year Papanicolaou Test

Variable	*F* test	*P* value^a^	Unadjusted (n = 3706; weighted n = 188 243 531)		*F* test	*P* value^a^	Adjusted (n = 3501; weighted n = 180 697 589)^b^	
			OR (95% CI)	*P* value^c^			OR (95% CI)	*P* value^c^
Rural vs urban residence	0.5	.49	0.85 (0.52-1.38)	.50	<0.1	.88	0.96 (0.56-1.65)	.88
Survey year								
2020 vs 2019	3.9	.02	0.93 (0.68-1.27)	.64	3.4	.04	0.94 (0.67-1.32)	.70
2022 vs 2019			0.70 (0.53-0.93)	.01			0.70 (0.52-0.95)	.02
Residence by survey year								
Rural: 2020 vs 2019	1.1	.33	0.67 (0.30-1.50)	.32	1.2	.30	0.64 (0.29-1.40)	.26
Rural: 2022 vs 2019			0.63 (0.34-1.19)	.15			0.58 (0.28-1.20)	.14
Age, y								
21-29	NA	NA	NA	NA	4.7	.003	1 [Reference]	
30-39							1.34 (0.86-2.09)	.20
40-49							1.20 (0.79-1.83)	.38
50-59							0.91 (0.61-1.36)	.64
60-65							0.63 (0.41-0.98)	.04
Race and ethnicity^d^								
Asian	NA	NA	NA	NA	3.87	.008	0.62 (0.28-1.39)	.24
Black							1.89 (1.31-2.72)	.001
Hispanic							1.20 (0.83-1.73)	.33
White							1 [Reference]	
Other^e^							0.79 (0.37-1.67)	.52
Partnered vs nonpartnered	NA	NA	NA	NA	15.67	<.001	1.61 (1.26-2.04)	<.001
Insured vs uninsured	NA	NA	NA	NA	46.50	<.001	3.82 (2.58-5.68)	<.001
Educational level								
<High school	NA	NA	NA	NA	4.18	.01	1 [Reference]	
High school graduate							1.14 (0.67-1.96)	.62
Some college							1.22 (0.70-2.15)	.47
≥College graduate							1.84 (1.07-3.18)	.03

Abbreviations: NA, not applicable; OR, odds ratio.

^a^ Calculated from overall *F* test of fixed effects.

^b^ Only those with complete data on all variables were included in the adjusted model.

^c^ Calculated from unpaired, 2-tailed *t* test of coefficient.

^d^ Race and ethnicity were self-reported and obtained from the Health Information National Trends Survey data.

^e^ Other includes non-Hispanic American Indian or Alaskan Native, non-Hispanic Native Hawaiian or Other Pacific Islander, and non-Hispanic multiple races.

The other sociodemographic characteristics associated with past-year Papanicolaou screening were age (*F* = 4.7; *P* = .003), race and ethnicity (*F* = 3.87; *P* = .008), partnered status (*F* = 15.67; *P* < .001), insurance coverage (*F* = 46.50; *P* < .001), and educational level (*F* = 4.18; *P* = .01). Compared with younger participants (21-29 years), those aged 60 to 65 years had 37% reduced odds of screening in the past year (OR, 0.63; 95% CI, 0.41-0.98; *P* = .04). Participants who reported having Black race and ethnicity had 89% increased odds of past-year Papanicolaou testing compared with White individuals (OR, 1.89; 95% CI, 1.31-2.72; *P* = .001). Partnered participants compared with single participants or those not living with a partner had 61% higher odds of past-year Papanicolaou testing (OR, 1.61; 95% CI, 1.26-2.04; *P* < .001), and participants with insurance had nearly a 4-fold increase in the odds of past-year Papanicolaou screening compared with those without insurance (OR, 3.82; 95% CI, 2.58-5.68; *P* < .001). Participants with a college degree vs those with less than a high school diploma had nearly a 2-fold increase in odds of past-year Papanicolaou screening (OR, 1.84; 95% CI, 1.07-3.18; *P* = .03).

## Discussion

This study’s findings provide further evidence that the COVID-19 pandemic disrupted the receipt of a major form of cervical cancer screening, Papanicolaou testing. Past-year Papanicolaou testing rates did not differ between 2019 and 2020, but they were significantly lower in 2022 than in 2019. The public health implications of delayed or missed screenings are worrisome because delays could be a factor in increased cervical cancer incidence, more advanced disease stage at diagnosis, and higher mortality in the future. One study modeled the long-term outcomes of delayed cervical cancer screenings and found that decreased screenings due to pandemic disruptions could be associated with a slight increase in the incidence of cervical cancer.^2,21^ On the other hand, the lower rates of past-year Papanicolaou tests in 2022 compared with 2019 may have been overestimated, as prior research has indicated the overuse of Papanicolaou tests among females.^14^

Unadjusted past-year Papanicolaou testing rates were significantly lower among rural than urban females. Although a rural vs urban difference did not persist in the adjusted logistic models, the unadjusted difference raised concerns that rural residents may be particularly susceptible to future cervical cancer incidence. Exactly why rural residents were less likely to receive a recent Papanicolaou test was unclear, but prior research lends some potential explanations. As noted, the availability of Papanicolaou testing at Medicare-designated rural health clinics decreased during the pandemic.^11^ Another study found that COVID-19 testing policies restricted access to cancer screenings in rural primary care clinics in particular.^12^ Accessibility has undoubtedly improved over time, but long-standing barriers to cervical cancer screening may remain for many rural females. A 2021 qualitative study of public health nurses in rural areas found that common barriers to cervical cancer screening include insufficient access to health care practitioners because of health care facility closures or consolidations; clinician shortages; lack of available appointments; and financial burdens, such as lack of insurance and underinsurance, inability to afford copayments, and/or inability to afford or access transportation to appointments.^22^ Other research suggests that the supply of primary care professionals serves as a mediator of the association between rural residence and cervical cancer.^23^ To facilitate an increase in Papanicolaou testing among rural residents, clinics may need to implement systems to remind clinicians and patients of their screening histories, increase the availability of clinic appointments, and accommodate residents’ schedules by offering screenings during evening or weekend hours or through mobile clinics.

In addition, the present study’s findings point to a need to target females aged 60 to 65 years and those without health insurance in promoting cervical cancer screening. Individuals in this age range may need to be reminded that they need a Papanicolaou test, assuming that they have not received a high-risk HPV test within the recommended time frame. The association between insurance coverage and a past-year Papanicolaou test underscores the importance of ensuring financial access. The number of persons without insurance increased slightly between the spring and winter of 2020,^24^ but losses of employer-sponsored insurance were offset by enrollment in Medicaid between 2019 and 2020.^25^ Continued increases in insurance coverage, whether through Medicaid expansions or Affordable Care Act Health Insurance Marketplace enrollment assistance programs,^26^ could be a factor in the receipt of cervical cancer screenings.

### Strengths and Limitations

The study methods presented some limitations. Because HINTS is a series of cross-sectional surveys, the findings can be used to examine population patterns over time, but no inferences can be made about longitudinal changes in individuals’ cancer screenings. Moreover, the exclusion from HINTS of high-risk HPV testing, which is a component of recommended cervical cancer screening for females 30 to 65 years of age,^13^ could have led to the underrepresentation of overall screening.

Overall, we believe these limitations are outweighed by the study’s strengths, including the recency of the data, repeated cross-sectional surveys, and nationally representative estimates for individuals regardless of their health insurance coverage. In contrast, other research examining rural and urban variations in cervical cancer screening rates relied on administrative data for individuals receiving services from a particular program in 2020.^4^

## Conclusions

Papanicolaou testing rates decreased markedly during the COVID-19 pandemic. Delayed or missed screenings may be a factor in increased incidence of cervical cancer and more advanced stages of disease at diagnosis in the future. Health care organizations, especially those serving rural females, should consider, at least temporarily, expanding access to Papanicolaou tests to increase cervical cancer screening rates to prepandemic levels.

## Notes

Supplement.
Data Sharing Statement
