# Supplementary material for: Cervical Cancer Screening Rates Among Rural and Urban Females, From 2019 to 2022
Source: JAMA Netw Open. 2024 Jun 14;7(6):e2417094. doi: 10.1001/jamanetworkopen.2024.17094 (PMC11179126; doi:10.1001/jamanetworkopen.2024.17094)
Supplement: Supplement. — Data Sharing Statement [file jamanetwopen-e2417094-s001.pdf]

Data Sharing Statement

Borders. Cervical Cancer Screening Rates Among Rural and Urban Females, From 2019 to 2022. *JAMA Netw Open*. Published June 14, 2024. doi:10.1001/jamanetworkopen.2024.17094

Data
Data available: Yes
Data types: Data (not involving human participants)
How to access data: The HINTS data are in the public domain at https://hints.cancer.gov/data/Default.aspx
When available: With publication

Supporting Documents
Document types: None

Additional Information
Who can access the data: The HINTS data are in the public domain.
Types of analyses: We do not plan to supply the analysis code.
Mechanisms of data availability: The data are in the public domain.
